# Supplementary material for: Transcriptomic Insights into Mechanisms of Early Seed Maturation in the Garden Pea (Pisum sativum L.)
Source: Cells. 2020 Mar 23;9(3):779. doi: 10.3390/cells9030779 (PMC7140803; doi:10.3390/cells9030779)

Zhewan−1,10(1)

Global Summary

%DE = 0.26

genes with fdr < 0.2 = 151 (68 + / 83 −)

genes with fdr < 0.1 = 151 (68 + / 83 −)

genes with fdr < 0.05 = 147 (67 + / 80 −)

genes with fdr < 0.01 = 127 (57 + / 70 −)

genes in genesets = 0

<FC> = 0

<t−score> = 0

<p−value> = 0

<fdr> = 0.74

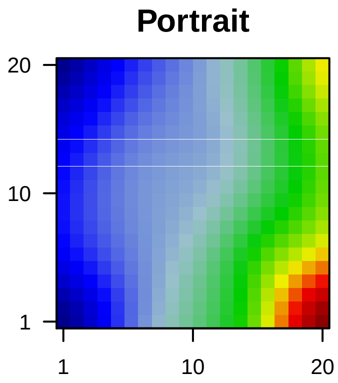

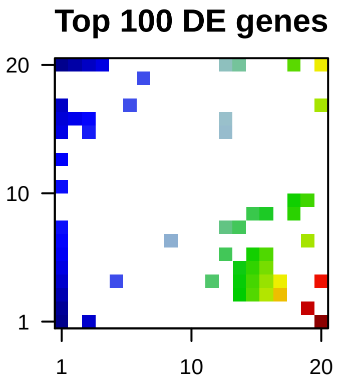

Global Genelist

Rank	ID	log(FC)	p−value	fdr	Metagene	Description
1	TRINITY_DN1	−0.29	2e−16	2e−15	1 x 2	
2	TRINITY_DN1	5.71	2e−16	2e−15	20 x 1	
3	TRINITY_DN1	3.01	2e−16	2e−15	20 x 20	
4	TRINITY_DN5	−0.32	2e−16	2e−15	2 x 20	
5	TRINITY_DN3	−0.18	2e−16	2e−15	1 x 15	
6	TRINITY_DN5	−0.23	2e−16	2e−15	1 x 11	
7	TRINITY_DN5	0.44	2e−16	2e−15	15 x 6	
8	TRINITY_DN2	1.04	2e−16	2e−15	20 x 1	
9	TRINITY_DN2	0.61	2e−16	2e−15	20 x 1	
10	TRINITY_DN3	0.36	2e−16	2e−15	20 x 17	
11	TRINITY_DN5	−0.47	2e−16	2e−15	1 x 1	
12	TRINITY_DN3	0.2	2e−16	2e−15	16 x 9	
13	TRINITY_DN5	0.26	2e−16	2e−15	14 x 5	
14	TRINITY_DN5	0.23	2e−16	2e−15	14 x 4	
15	TRINITY_DN4	0.26	2e−16	2e−15	15 x 3	
16	TRINITY_DN4	−0.9	2e−16	2e−15	1 x 1	
17	TRINITY_DN4	−0.49	2e−16	2e−15	18 x 20	
18	TRINITY_DN1	−0.17	2e−16	2e−15	1 x 1	
19	TRINITY_DN3	0.57	2e−16	2e−15	20 x 1	
20	TRINITY_DN2	−0.19	2e−16	2e−15	1 x 2	

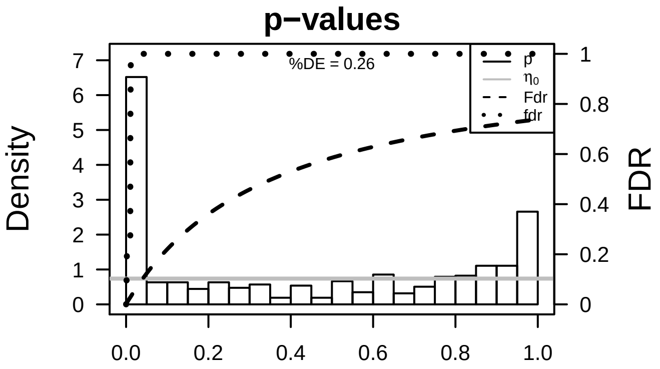

Supplement: Supplementary file 1 [file cells-09-00779-s001.zip › Supplementary materials/FileS3.zip!/FileS2/Hormones - Results/Summary Sheets - Samples/Zhewan.1.10.1..pdf]
